# First reported case of *Staphylococcus condimenti* infection associated with catheter-related bacteraemia

**DOI:** 10.1016/j.nmni.2014.10.002

**Published:** 2014-10-17

**Authors:** Y. Misawa, A. Yoshida, S. Okugawa, K. Moriya

**Affiliations:** 1) Department of Infection Control and Prevention, the University of Tokyo Hospital, Tokyo, Japan; 2) Department of Infection Control, Dokkyo Medical University Hospital, Mibu, Shimotsuga-gun, Tochigi, Japan

**Keywords:** Catheter-related bacteraemia, coagulase test, latex agglutination test, lecithinase and lipase reaction, *Staphylococcus condimenti*

## Abstract

We report a case of a patient who experienced a catheter-related bloodstream infection caused by *Staphylococcus condimenti*, which was first isolated from soy sauce mash. This is the first reported case of human infection. Although blood culture isolates and the catheter tip tube did not reveal coagulase or clumping factor, false-positive results were obtained from latex agglutination tests for clumping factor and protein A due to self-agglutination. Care is needed when performing only latex agglutination test without a coagulase test. Further studies are needed to determine the pathogenic potential of *S. condimenti* based on appropriate identification.

Coagulase-negative staphylococci are found among skin commensals and are regarded as less pathogenic than coagulase-positive staphylococci, such as *Staphylococcus aureus*. However, coagulase-negative staphylococci have been increasingly found to cause significant nosocomial infections, such as catheter-related bloodstream infections, prosthetic valve endocarditis, and central nervous system shunt infections and prosthetic joint infections [1,2].

Currently, more than 40 species of coagulase-negative staphylococci have been identified [3]. Although accurate identification has become increasingly important to define the clinical significance of these coagulase-negative staphylococci and to achieve better management of patients, identification to species level is often difficult, even when using 16S rRNA sequencing [4].

*Staphylococcus condimenti* was first isolated from soy sauce mash and suggested to be a new species in 1998 [5]. However, it has never been reported as a human pathogen, and its microbiological characteristics have not been fully elucidated. Here, we report a case of *S. condimenti* bloodstream infection and its bacteriological characteristics.

A 17-year-old female patient with severe dilated cardiomyopathy was implanted with a left ventricular assist device. She developed a high fever (39°C) with no specific focal signs of infection 5 months after a central vein catheter was inserted from the right internal jugular vein. Because catheter-related bloodstream infection was suspected, the central venous catheter was removed, and two sets of blood cultures (BacT/ALERT 3D system, SYSMEX bioMérieux, Durham, NC, USA) and the catheter tip were sent to the microbiology laboratory (Day 0). Antibiotic therapy using piperacillin/tazobactam (4.5 g three times a day) was initiated, and the patient's fever began to decrease within 24 h. The next day, intravenous vancomycin (1 g twice daily) was added because one set of blood cultures and the catheter tip were positive for clusters of gram-positive cocci. After this, the patient's condition stabilized, and piperacillin/tazobactam and vancomycin were switched to cefazolin (1 g four times a day) for a further 6 days. The patient's condition improved uneventfully and the results of subsequent blood cultures (Days 4 and 23) remained negative.

One set of blood cultures obtained on Day 0 yielded a positive result in both aerobic and anaerobic bottles after 18 h of incubation. In subsequent cultures on 5% sheep blood agar at 35°C with 5% CO_2_ gas, white, circular, bulging colonies began to grow without haemolysis and reached 1–2 mm in diameter after 24 h. Both isolates (TD8891 from blood, and TD6610 from the catheter) were catalase positive, mannitol positive, lecithinase reaction weakly positive, and lipase reaction negative on mannitol salt agar with egg yolk (NISSUI Pharmaceutical, Tokyo, Japan) after 48 h of incubation. Although a tube coagulase test using rabbit plasma (Eiken Chemical, Tokyo, Japan) was negative for both isolates, lumps resembling small fibrin were observed. Moreover, in the latex agglutination assay (Denka Seiken, Tokyo, Japan), which contains clumping factor and protein A, strong agglutination was shown whereas when colonies were mixed in saline only, those strains showed clear self-agglutination. Furthermore, upon examination with the Pastorex Staph Plus test (BioRad, Marnes-la-Coquette, France), both isolates and the negative control showed positive results.

A VITEK 2 instrument using the gram-positive card (SYSMEX bioMérieux) identified the isolates as *Staphylococcus carnosus* subsp. *carnosus* with a probability of 95%, and Microscan Pos Combo Panel 3.1J (SIEMENS Healthcare Diagnostics, Tokyo, Japan) showed *Staphylococcus xylosus* (TD8891) with a probability of 43% and *Staphylococcus intermedius* (TD6610) with a probability of 56%. The results of these conventional methods were not in agreement. We, therefore, conducted *hsp60* and *sodA* gene sequencing [4,6]. In both isolates, a BLAST search showed 100% concurrence with the sequence of *Staphylococcus condimenti* CCUG 39902^T^ (DSM 11674, Accession No. AJ343904), suggesting that the isolates were *S. condimenti*. The biochemical characteristics were comparable to the *S. condimenti* type strain (CCUG 39902^T^), TD8891 and TD6610 using VITEK2 (Table 1). *Sma*I-digested pulsed-field gel electrophoresis showed identical patterns for the two strains (Fig. 1), indicating that one strain caused the catheter-related bloodstream infection. Minimal inhibitory concentrations of antimicrobial agents were determined by broth microdilution methods, and the minimal inhibitory concentrations of each antimicrobial agent were evaluated according to the latest EUCAST documents. Both isolates were susceptible to penicillin, oxacillin, cefazolin, levofloxacin, amikacin, vancomycin, teicoplanin, erythromycin, clarithromycin, clindamycin, minocycline, daptomycin, linezolid and mupirocin.

This case is the first to show the pathogenic potential of *S. condimenti* as the causative bacteria in a catheter-related bloodstream infection, and the expected virulence of *S. condimenti* may be very low [7]. However, the bacteria may be a conditional pathogen. Considerating that its first isolation was from soy sauce mash [5], *S. condimenti* may exist in the environment in Japan, where soy sauce is frequently used on a daily basis.

In all isolates, strong self-agglutination was observed, but a small fibrin lump was observed in the tube coagulase test; this suggests that *S. condimenti* shows a propensity towards self-agglutination. *Staphylococcus lugdunensis*, *Staphylococcus schleiferi*, *S. intermedius* and *Staphylococcus hyicus* may react with Pastorex Staph Plus as the clumping factor and fibrinogen affinity factor because Pastorex Staph Plus is made from latex particles sensitized by fibrinogen and IgG, as well as specific monoclonal antibodies against capsular polysaccharides of *S. aureus*. Additionally, some strains of Staphylococcus, particularly *Staphylococcus saprophyticus*, are known to cause non-specific aggregation of latex particles [8]. However, there are no reports of *S. condimenti* showing a non-specific aggregation reaction.

Latex tests are often used in lieu of tube coagulase tests in many institutions because they favourably correlate with tube coagulase test results [9] and can quickly confirm a reaction. However, we identified *S. condimenti* as a result of inconsistencies between these two tests. If only a latex agglutination assay is used, then *S. aureus* or coagulase-positive staphylococci may be incorrectly reported. To differentiate *S. condimenti* from *S. aureus*, lipolytic activity on egg yolk agar including lecithinase and lipase reactions are useful: *S. aureus* shows positive results in these two reactions, whereas *S. condimenti* exhibits a weak lecithinase reaction and no lipase reaction.

We conclude that *S. condimenti* can cause false-positive and misleading results when using rapid agglutination kits with regard to the identification of *S. aureus*. Other verification tests, such as tube coagulase and egg yolk reaction tests should be carried out to properly identify *S. condimenti*.

## Conflict of interest

There were no conflicts of interest in this study.

## Figures and Tables

**FIG. 1:**
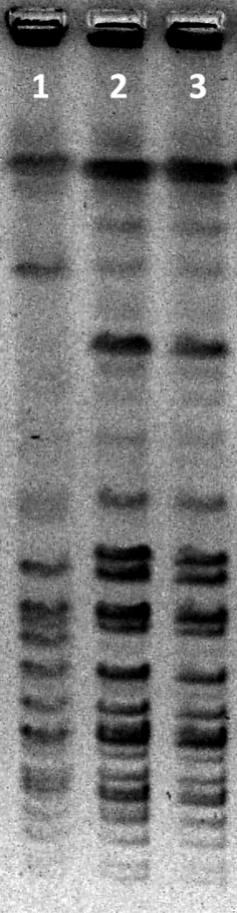
*Sma*I digests showing the following three pulsed field gel electrophoresis types: lane 1, CCUG39902; lane 2, TD8891; and lane 3, TD6610.

**TABLE 1:** Characteristics of *Staphylococcus condimenti* CCUG 39902^T^ and clinical isolates^a^

Characteristic	CCUG 39902^T^	TD8891	TD6610
Manual tests results:			
Catalase	+	+	+
Tube coagulase test	—	—	—
Latex agglutination test^b^			
Negative control	—	+	+
Latex test	—	+	+
Mannitol fermentation for 24 h	—	w	w
Mannitol fermentation for 48 h	+	+	+
Egg yolk reaction			
Lecithinase reaction for 24 h	—	—	—
Lecithinase reaction for 48 h	w	w	w
Lipase reaction for 48 h	—	—	—
VITEK2 results:			
Ala-Phe-Pro Arylamidase	—	—	—
Alanine arylamidase	—	—	—
l-Aspartate arylamidase	—	—	—
l-Proline arylamidase	—	—	—
l-Pyrrolidonyl-arylamidase	—	—	+
Leucine arylamidase	—	—	—
Tyrosine arylamidase	—	—	—
α-Galactosidase	—	—	—
α-Glucosidase	—	—	—
α-Mannosidase	—	—	—
β-Galactopyranosidase	—	—	—
β-Galactosidase	+	+	+
Nitrophenyl-β-glucuronidase	—	—	—
Resorufin-β-glucuronidase	—	—	—
Phosphatase	+	+	+
Phosphatidylinositol phospholipase C	—	—	—
Arginine dihydrolase 1	+	+	+
Arginine dihydrolase 2	+	+	+
Urease	+	+	+
Growth in 6.5% NaCl	+	+	+
l-Lactate alkalinization	—	+	+
Cyclodextrin	—	—	—
d-Amygdalin	—	—	—
d-Galactose	—	—	—
d-Maltose	—	—	—
d-Mannitol	+	+	+
d-Mannose	+	+	+
d-Raffinose	—	—	—
d-Ribose	—	—	—
d-Sorbitol	+	+	+
d-Trehalose	+	+	+
d-Xylose	—	—	—
Lactose	+	+	+
Methyl-B-d-glucopyranoside	—	—	—
*N*-Acetyl-d-glucosamine	+	—	—
Pullulan	—	—	—
Salicin	—	—	—
Sucrose	—	—	—
Bacitracin resistance	—	+	+
Novobiocin resistance	+	—	+
O/129 resistance	+	+	+
Optochin resistance	+	+	+
Polymyxin B resistance	+	—	—

a +, positive; —, negative; w, weakly positive.

b Results of Pastorex Staph Plus.
